# Fall-Related Injuries in Children Aged 0–6 Years: A Trauma Registry Study in Riyadh, Saudi Arabia

**DOI:** 10.3390/jcm15208104

**Published:** 2026-10-19

**Authors:** Rayan Jafnan Alharbi

**Affiliations:** Department of Basic Health Sciences, College of Applied Medical Sciences, Qassim University, Saudi Arabia; r.alharbi14@qu.edu.sa

**Keywords:** paediatric falls, injury prevention, injury severity, head injury, intensive care

## Abstract

**Background/Objectives**: Falls are a leading cause of injury in early childhood, but detailed trauma registry data from Saudi Arabia remain limited. This study aimed to describe the demographic profile, injury mechanisms, and determinants of severity among trauma registry-enrolled children aged 0–6 years with fall-related injuries treated at a major trauma centre in Riyadh. **Methods**: A single-centre retrospective cohort study was conducted using the Saudi TraumA Registry at King Saud Medical City, a major tertiary trauma centre in Riyadh. Children aged 0–6 years with fall-related injuries meeting the registry inclusion criteria between 1 August 2017 and 31 December 2022 were included. Demographic, injury and clinical variables were extracted and summarised using descriptive statistics. Univariate logistic regression identified several factors associated with ICU admission and mechanical ventilation; variables significant at *p* < 0.05 were entered into multivariable logistic regression models to identify independent associations with the primary outcomes. **Results**: A total of 258 trauma registry-enrolled children aged 0–6 years with fall-related injuries were included (male n = 172, 66.7%; Saudi nationals n = 212, 82.2%). Falls from heights > 1 m were most frequent (n = 153, 59.3%), and the home was the most common setting (n = 157, 60.9%). The most frequent injury types were upper-extremity injuries (n = 114, 42.2%) and head injuries (n = 77, 28.5%). Two-thirds of children underwent an operation (n = 172, 66.7%); 39 were admitted to intensive care (15.1%), and 25 required mechanical ventilation (9.7%). In multivariable analysis, head injury was independently associated with higher odds of ICU admission (adjusted OR 6.4, 95% CI 1.99–20.6; *p* = 0.002) and mechanical ventilation (adjusted OR 17.6, 95% CI 3.42–91.0; *p* < 0.001). Female patients had significantly higher adjusted odds of ICU admission (AOR 3.13, 95% CI 1.22–8.04; *p* = 0.018). Thoracic injury was independently associated with mechanical ventilation (adjusted OR 13.3, 95% CI 1.67–106.7; *p* = 0.014). In-hospital mortality was 1.2% (n = 3). **Conclusions**: Among trauma registry-enrolled children with fall-related injuries presenting to a major trauma centre in Riyadh, most had low anatomical injury severity based on ISS; however, a meaningful minority, particularly those with head or thoracic injuries, required operative management or critical care. These findings highlight the importance of injury prevention and early recognition of children at increased risk of severe outcomes following a fall.

## 1. Introduction

Falls among young children are a major public-health concern worldwide because of their frequency and potential for serious harm [1,2]. Children aged 0–6 years are especially vulnerable because rapid physical development, limited hazard awareness and immature motor coordination increase their risk of injury [3,4]. Internationally, falls rank among the leading causes of paediatric emergency department attendances and injury-related hospital admissions, frequently resulting in head trauma, fractures and, in some cases, long-term functional impairment [5,6].

In Saudi Arabia, paediatric falls account for a substantial proportion of injury-related healthcare presentations [7,8], yet detailed epidemiological data on their characteristics, mechanisms and severity, especially from large urban centres such as Riyadh, remain limited. A clearer, locally specific understanding of these events is needed to develop targeted prevention measures, inform public-health actions and reduce both the immediate and long-term harms of fall-related injury in early childhood.

Several factors influence both the frequency and severity of childhood falls, including age-specific developmental milestones, environmental hazards, caregiver supervision and socioeconomic determinants [9]. Younger children typically sustain falls from furniture or other elevated surfaces within the home, reflecting developmental behaviours such as crawling, climbing and exploratory play [10]. By contrast, older preschoolers more frequently sustain falls from greater heights, playground equipment, windows, stairs or outdoor structures, consistent with increased mobility and more independent exploration [11,12].

Socioeconomic circumstances, rural residence, low parental education and household safety practices further influence both the occurrence and severity of childhood falls [13,14]. Despite international evidence identifying these drivers, detailed local data that capture the specific mechanisms and outcomes of paediatric falls in Saudi Arabia remain limited [15]. Region-specific evidence is therefore needed to inform pragmatic, culturally appropriate prevention measures and to guide caregiver education. This study aimed to provide a comprehensive examination of fall-related injuries among trauma registry-enrolled children aged 0–6 years presenting to a major trauma centre in Riyadh, characterising demographic profiles and injury mechanisms, describing clinical outcomes and identifying factors associated with severe injury requiring critical care.

## 2. Materials and Methods

This study employed a retrospective cohort design using routinely collected trauma data from King Saud Medical City (KSMC), a tertiary referral and major trauma centre in Riyadh. Cases were extracted from the Saudi TraumA Registry (STAR) for the period 1 August 2017 to 31 December 2022. STAR prospectively records clinical and administrative information for patients meeting standard trauma-entry criteria at KSMC; for this analysis, all registry entries for children aged 0–6 years with a documented fall mechanism were eligible. Patients are entered into the registry if they meet at least one of the following criteria: principal diagnosis of injury; death in the Emergency Department (ED) attributable to the injury; an inpatient stay of three or more calendar days; in-hospital death following the injury; or admission to an Intensive Care Unit (ICU).

The analysis extracted a broad set of patient- and injury-level variables, including demographics (age, sex, nationality), injury characteristics (mechanism, anatomical site and location), mode of arrival to definitive care, and physiological measures on ED arrival (systolic blood pressure, heart rate, respiratory rate, oxygen saturation [SpO_2_]). Injury types were recorded according to the anatomical regions involved. Injury types were summarised at the patient level. As some children sustained injuries involving more than one anatomical region, injury categories were not mutually exclusive. Percentages for each injury type were calculated using the total number of patients (n = 258) as the denominator. Falls were classified as low-level/same-level falls or falls from a height > 1 metre based on the mechanism and estimated fall height documented in the STAR. Fall height was based on the clinical history recorded at presentation rather than objective measurement. Level of consciousness was recorded using the Glasgow Coma Scale (GCS) and injury severity using the Injury Severity Score (ISS).

Key clinical outcomes included ICU admission, requirement for surgery, mechanical ventilation and in-hospital mortality. ICU admission was defined as any admission to an intensive care unit during the hospital stay. Mechanical ventilation was defined as invasive ventilatory support recorded in the trauma registry, irrespective of whether ICU admission occurred directly from the emergency department or later during hospitalisation. The variable requiring operation was defined according to the STAR as any therapeutic procedure performed in the operating theatre as part of the treatment provided to a trauma patient at KSMC. Operative management was included only in the univariate analysis as a treatment-related variable reflecting healthcare utilisation, treatment intensity, and injury-management complexity during hospitalisation. It was not included in the multivariable models. The original age categories were retained to preserve age-specific differences in fall mechanisms and injury patterns during early childhood and to avoid obscuring potentially relevant variation across individual ages. ISS and GCS were categorised using established clinical thresholds [16], while mechanical ventilation duration was categorised according to the STAR definitions.

Data were summarised using descriptive statistics: categorical variables as counts and percentages, and continuous variables as mean ± standard deviation (SD) or median (interquartile range [IQR]), depending on distribution. Between-group comparisons across age bands used chi-square tests for categorical variables and one-way ANOVA or Kruskal–Wallis tests for continuous variables, as appropriate.

Univariate logistic regression was used to examine associations between potential factors and the two primary outcomes (ICU admission and mechanical ventilation). Variables with a *p*-value < 0.05 in univariate analysis, together with clinically relevant covariates identified a priori, were considered for multivariable logistic regression. Clinically relevant variables were selected based on their established relationship with injury severity and the outcomes of interest. Multicollinearity among candidate covariates was assessed before model development, and variables demonstrating substantial clinical overlap with the study outcomes or other injury-severity variables, including Injury Severity Score (ISS) and Glasgow Coma Scale (GCS), were excluded from the final models to minimise multicollinearity. Adjusted odds ratios (AORs) with 95% confidence intervals were reported to estimate independent associations with the two primary outcomes. Analyses were performed using complete-case data, with observations containing missing values excluded from the respective regression models. All statistical tests were two-sided, and a *p*-value < 0.05 was considered statistically significant. Statistical analyses were performed using IBM SPSS Statistics for Windows, version 30.0. This study was approved by the Institutional Review Board at King Saud Medical City (Reference No. H1RI-02-Mar25-05). All procedures were conducted in accordance with the ethical standards of the 1964 Declaration of Helsinki and its later amendments.

## 3. Results

A total of 258 children aged 0–6 years were included. The largest age group was 6 years (n = 44, 17.1%), followed by 1-year-olds (n = 42, 16.3%). Most patients were male (n = 172, 66.7%) and Saudi nationals (n = 212, 82.2%). Injuries occurred predominantly at night (n = 195, 75.6%) and more frequently on weekdays (n = 186, 72.1%). Falls from heights > 1 metre were more common than low-level falls (n = 153, 59.3%). Injury location varied by age group, with the home being the most frequent setting overall (n = 157, 60.9%; *p* = 0.001).

Regarding injury patterns and arrival characteristics, the two most frequent injury types were upper-extremity injuries (n = 114, 42.2%) and head injuries (n = 77, 28.5%). Younger children had a higher proportion of head injuries, with more than half of infants aged <1 year (n = 10, 55.6%) and 1-year-olds (n = 23, 53.5%) sustaining head trauma, compared with 13.7% of six-year-olds (n = 7; *p* = 0.001). Arrival to definitive care was most commonly by private/police vehicle (n = 148, 57.4%), followed by government/private ambulance (n = 59, 22.9%). Mode of arrival also varied by age, for example, two-year-olds more commonly presented via private/police vehicle (n = 29, 76.3%) than infants (n = 12, 52.2%).

**Figure 1:**
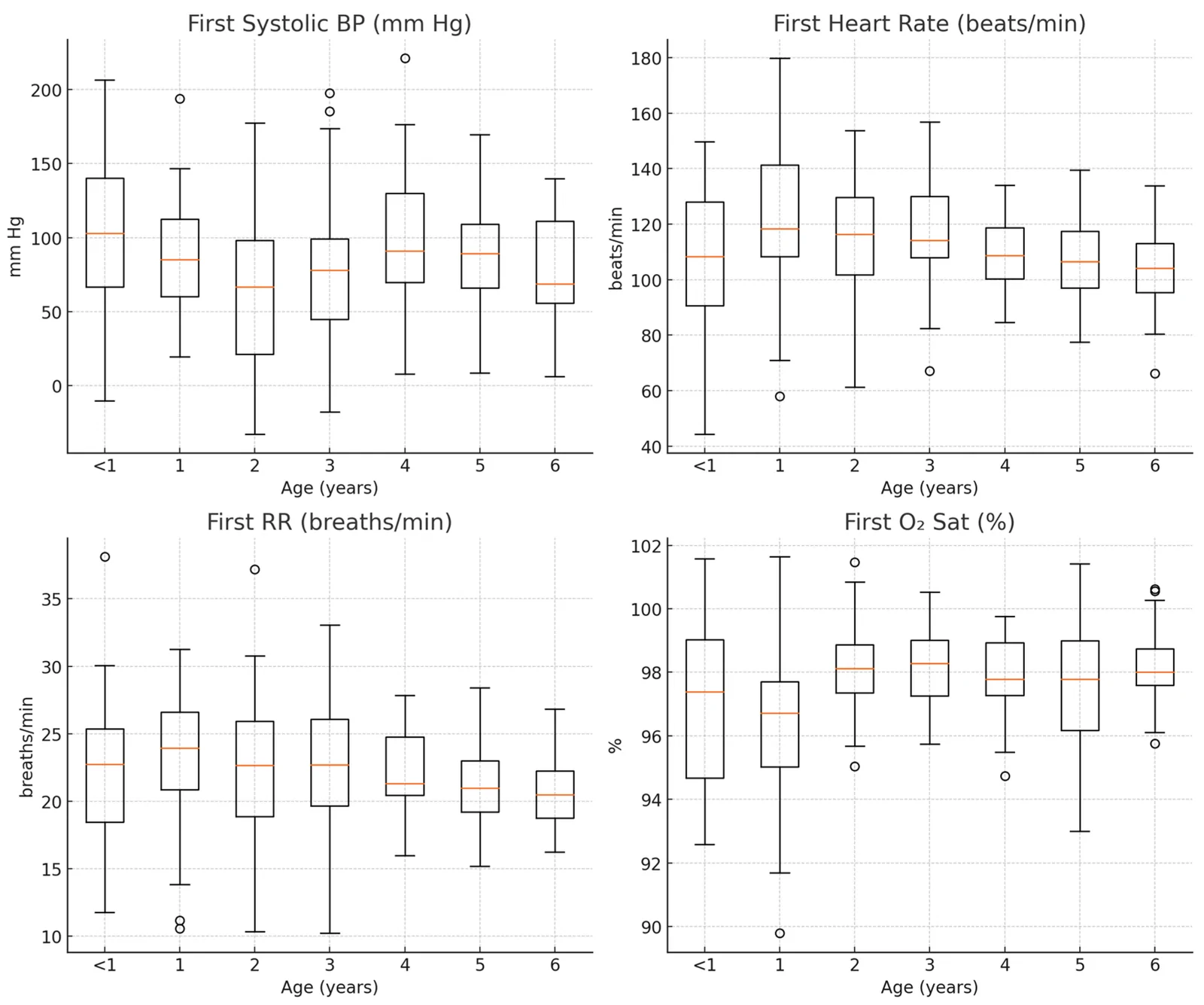
Distribution of initial physiologic measures including first systolic blood pressure (mm Hg), heart rate (beats/min), respiratory rate (breaths/min), and oxygen saturation (%) across paediatric age groups on arrival at the ED.

The majority of children (n = 186, 73.2%) presented directly to KSMC, whereas 68 (26.8%) were transferred from another healthcare facility prior to presentation. Trauma team activation was uncommon (n = 7, 2.7%), and blood transfusion in the ED was required in only seven patients (2.7%) (Table 1). On arrival to the ED, the mean systolic blood pressure was 89 mmHg (SD 42.4), mean heart rate was 112 bpm (SD 21.3), mean respiratory rate was 22 breaths/min (SD 4.0), and mean oxygen saturation was 97% (SD 1.9) (Figure 1).

**Figure 2:**
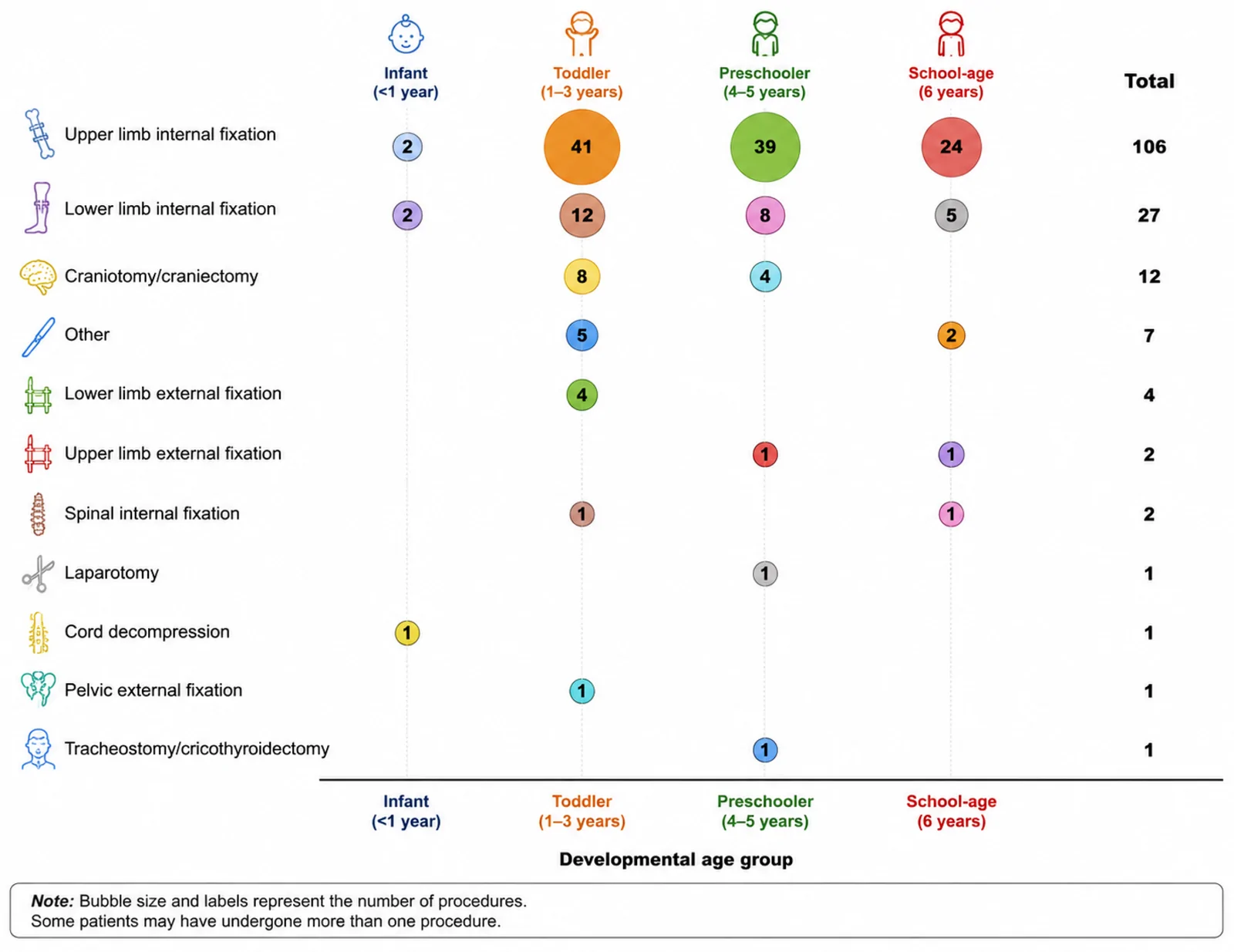
Distribution of surgical procedures by developmental age group among children aged 0–6 years with fall-related injuries.

Table 2 summarises level of consciousness, injury severity and clinical outcomes. Most children presented with normal consciousness (GCS 13–15: n = 201, 77.9%). Infants were less likely to present with GCS 13–15 (n = 15, 65.2%) than six-year-olds (n = 39, 88.6%). Injury severity was generally low, with 88.8% of children having an ISS ≤ 15 (n = 229). Most patients were admitted to the ward (n = 217, 84.1%), while 39 children (15.1%) required ICU admission. Two-thirds of patients underwent surgical intervention (n = 172, 66.7%), although this proportion was lower among infants (n = 9, 39.1%) than among six-year-olds (n = 34, 77.3%). Figure 2 shows the distribution of surgical procedures by developmental age group. Upper limb internal fixation was the predominant procedure across all age groups, followed by lower limb internal fixation, whereas all other procedures were relatively uncommon. Mechanical ventilation was required in 25 patients (9.7%). Median hospital length of stay was 3.0 days (IQR 3), with a mean stay of 5.7 days; mean ICU stay was 1.3 days. Most patients were discharged home (n = 253, 98.1%), and in-hospital mortality was low (n = 3, 1.2%).

Univariate logistic regression identified several factors significantly associated with mechanical ventilation and ICU admission (Table 3). For mechanical ventilation, significant univariate associations were observed with falls from >1 metre (OR 2.8, 95% CI 1.2–6.7; *p* = 0.016), head injury (OR 23.7, 95% CI 6.8–82.3; *p* < 0.001), thoracic injury (OR 9.4, 95% CI 2.6–33.7; *p* = 0.001), arrival by helicopter (OR 20.1, 95% CI 1.7–231.0; *p* = 0.016) and arrival by government/private ambulance (OR 7.8, 95% CI 3.2–18.9; *p* < 0.001). Requirement for operative intervention was also associated with increased odds of mechanical ventilation (OR 5.0, 95% CI 2.0–12.2; *p* < 0.001). For ICU admission, factors significantly associated in the univariate analysis included female sex (OR 2.1, 95% CI 1.08–4.20; *p* = 0.029), fall from >1 metre (OR 2.7, 95% CI 1.3–5.4; *p* = 0.005), head injury (OR 14.5, 95% CI 6.2–33.8; *p* < 0.001), thoracic injury (OR 7.7, 95% CI 2.2–26.9; *p* = 0.001), spine injury (OR 6.0, 95% CI 1.1–30.8; *p* = 0.032), arrival by helicopter (OR 11.7, 95% CI 1.0–133.2; *p* = 0.046), arrival by government/private ambulance (OR 6.3, 95% CI 3.0–13.1; *p* < 0.001), and required operation (OR 4.0, 95% CI 1.9–8.2; *p* < 0.001).

In multivariate analysis, head injury was independently associated with higher odds of requiring mechanical ventilation (AOR 17.6, 95% CI 3.42–91.03; *p* < 0.001) and of ICU admission (AOR 6.4, 95% CI 1.99–20.64; *p* = 0.002). Female patients had significantly higher adjusted odds of ICU admission (AOR 3.13, 95% CI 1.22–8.04; *p* = 0.018). Thoracic injury was independently associated with mechanical ventilation (AOR 13.3, 95% CI 1.67–106.7; *p* = 0.014) and showed a non-significant trend towards increased odds of ICU admission (AOR 5.1, 95% CI 0.89–30.22; *p* = 0.067). No other variables retained statistical significance in the adjusted models (Table 4).

## 4. Discussion

This retrospective cohort study at a major trauma centre in Riyadh provides a detailed clinical profile of fall-related injuries among children aged 0–6 years. Most events occurred at home and involved elevated surfaces, and boys were over-represented. On univariate analysis, fall from height, head and thoracic injuries, arrival mode and the need for surgery were all associated with ICU admission and mechanical ventilation. After adjustment, head injury remained independently associated with higher odds of both outcomes, while thoracic injury was independently associated with higher odds of requiring mechanical ventilation. Although many presentations were of relatively low anatomical severity with preserved consciousness, a meaningful minority nevertheless required operative care, intensive-care admission or respiratory support, emphasising that common paediatric falls can still result in substantial morbidity. These findings highlight the need for focused prevention, timely triage, and strengthened surveillance to reduce serious fall-related harm in young children.

The predominance of home-based falls in this series aligns with patterns reported regionally and internationally, where the domestic setting is the most common scene for injuries in toddlers and preschoolers [17,18]. Previous studies report similar mechanisms—children climbing on furniture, tumbling from beds or windows, and losing balance on stairs or play equipment during unsupervised moments—which helps explain why younger age groups sustain many low-level falls that can nevertheless result in serious injury [17,18,19]. The male excess observed here is also widely reported and likely reflects differing play behaviours and greater exposure to risk [20]. Likewise, the association between greater fall height and a higher need for critical care in this cohort is consistent with prior reports linking fall distance to increased injury severity and worse outcomes [21]. These results underscore the importance of early identification of high-risk injuries and of prioritising home-based prevention measures, where most falls occur.

These results align with earlier research identifying head injury as the predominant and most clinically significant consequence of childhood falls [22,23,24,25]. In the present study, head injury was independently associated with higher odds of ICU admission and the need for mechanical ventilation, reflecting the clinical course of traumatic brain injury in which airway protection, intracranial pressure monitoring and close neurological observation are often required. Thoracic injuries, although less common, independently predicted need for ventilatory support, underscoring how chest-wall trauma and pulmonary contusion can rapidly impair respiratory mechanics in young children. Female sex was associated with ICU use in univariate analysis but did not retain significance in multivariable models. Overall, these findings support a risk-stratified approach in the emergency setting, in which mechanism and anatomical site guide early imaging, physiological monitoring and timely escalation to paediatric critical-care services.

Although most children had low anatomical injury severity based on ISS, two-thirds underwent operative management. This apparent discrepancy may be explained by the predominance of orthopaedic procedures, particularly upper and lower limb internal fixation. Upper limb internal fixation was the most frequently performed procedure across all developmental age groups, indicating that operative intervention was commonly required despite relatively low overall injury severity. These findings highlight that ISS reflects anatomical injury burden rather than the need for surgical management and underscore the considerable orthopaedic burden associated with fall-related injuries in young children treated at a tertiary trauma centre.

These findings have important implications for prevention and acute care. At the community level, prevention should focus on the home, for example, promoting simple, evidence-based measures such as window guards, stair gates and securing elevated furniture, alongside targeted education for parents and caregivers about supervision at key developmental stages [26,27,28]. Clinically, the observed associations suggest that injury mechanism and anatomical site, particularly head and thoracic injuries, should be considered during the assessment and risk stratification of children presenting with fall-related injuries. Prehospital and ED systems would benefit from clearer escalation criteria so that high-risk children are identified and transferred expediently to centres with appropriate trauma capability [29]. Finally, the results support expanding the trauma-registry model to additional hospitals and regions to strengthen regional surveillance and guide prevention and resource planning [30].

The study draws strength from analysis of a large, prospectively maintained trauma registry at a high-volume tertiary centre, which enabled systematic characterisation of injury mechanisms, physiological status on arrival and in-hospital interventions across a broad severity spectrum. Application of multivariable modelling improved understanding of factors independently associated with intensive-care use and ventilatory support.

Nonetheless, several limitations warrant consideration. As inclusion was restricted to children meeting STAR eligibility criteria, less severe fall injuries are likely to be underrepresented. Although most children (73.2%) presented directly to KSMC, the tertiary trauma centre setting and registry criteria may have contributed to greater injury complexity and the high operative rate. Therefore, the findings may not be generalizable to all paediatric falls in Riyadh or Saudi Arabia. In addition, the observational nature of registry data precludes causal inference. Several variables contained missing or undocumented data, particularly injury location, GCS, and mode of arrival. Although missing values were retained and reported in the descriptive analyses, complete-case analysis was used for the regression models. The mechanism of missingness could not be reliably determined, and the exclusion of incomplete cases may have introduced selection bias and reduced the precision of some estimates. Finally, the registry did not capture some contextual variables that may influence fall risk and care pathways, including detailed household circumstances and longer-term post-discharge outcomes. As the study was limited to in-hospital outcomes, long-term functional outcomes and complications could not be assessed, while small numbers for uncommon exposures resulted in imprecise estimates for some associations. These limitations highlight the value of multicentre collaborations and prospective follow-up studies to validate findings and assess longer-term outcomes.

## 5. Conclusions

This study provides a focused clinical account of fall-related injuries among trauma registry-enrolled children aged 0–6 years treated at a major trauma centre in Riyadh. While most children in this trauma registry cohort had low anatomical injury severity, head and thoracic injuries were independently associated with increased odds of ICU admission and mechanical ventilation. These findings provide insight into the characteristics and outcomes of young children with fall-related injuries requiring trauma centre care and support the need for further multicentre studies to validate these associations and better characterise injury patterns and long-term outcomes.

## Figures and Tables

**Table 1:** Descriptive characteristics of paediatric patients presenting with fall-related injuries (N = 258).

Variables	All Patients	Age < 1	1 Year	2 Years	3 Years	4 Years	5 Years	6 Years	*p*-Value
	N = 258	N = 23	N = 42	N = 38	N = 35	N = 36	N = 40	N = 44	
**Gender *n* (%)**									0.130
Male	172 (66.7)	20 (87.0)	25 (59.5)	21 (55.3)	25 (71.4)	21 (58.3)	28 (70.0)	32 (72.7)	
Female	86 (33.3)	3 (13.0)	17 (40.5)	17 (44.7)	10 (28.6)	15 (41.7)	12 (30.0)	12 (27.3)	
**Nationality *n* (%)**									0.326
Saudi	212 (82.2)	17 (73.9)	31 (73.8)	35 (92.1)	30 (85.7)	28 (77.8)	33 (82.5)	38 (86.4)	
Non-Saudi	46 (17.8)	6 (26.1)	11 (26.2)	3 (7.9)	5 (14.3)	8 (22.2)	7 (17.5)	6 (13.6)	
**Injury time, *n* (%)**									0.617
Day	63 (24.4)	7 (30.4)	12 (28.6)	8 (21.1)	7 (20.0)	5 (13.9)	11 (27.5)	13 (29.5)	
Night	195 (75.6)	16 (69.6)	30 (71.4)	30 (78.9)	28 (80.0)	31 (86.1)	29 (72.5)	31 (70.5)	
**Day of week, n (%)**									0.058
Weekday (Sun—Thur)	186 (72.1)	16 (69.6)	27 (64.3)	23 (60.5)	29 (82.9)	29 (80.6)	25 (62.5)	37 (84.1)	
Weekend (Fri—Sat)	72 (27.9)	7 (30.4)	15 (35.7)	15 (39.5)	6 (17.1)	7 (19.4)	15 (37.5)	7 (15.9)	
**Injury cause**									0.080
Fall (same level or <1 metre)	105 (40.7)	6 (26.1)	11 (26.2)	13 (34.2)	17 (48.6)	20 (55.6)	18 (45.0)	20 (45.5)	
Fall (>1 metre)	153 (59.3)	17 (73.9)	31 (73.8)	25 (65.8)	18 (51.4)	16 (44.4)	22 (55.0)	24 (54.5)	
**Injury Location, *n* (%)**									<0.001
Home/house	157 (60.9)	14 (60.9)	27 (64.3)	28 (73.7)	23 (65.7)	20 (55.6)	24 (60.0)	21 (47.7)	
Road	6 (2.3)	-	-	-	1 (2.9)	-	1 (2.5)	4 (9.1)	
School	4 (1.6)	1 (4.3)	-	-	1 (2.9)	-	1 (2.5)	1 (2.3)	
Industrial/construction	3 (1.2)	1 (4.3)	-	-	-	-	-	2 (4.5)	
Unknown	88 (34.1)	7 (30.4)	15 (35.7)	10 (26.3)	10 (28.6)	16 (44.4)	14 (35.0)	16 (36.4)	
Injury type, *n* (%) **									<0.001
Head	77 (29.8)	10 (43.5)	23 (54.8)	10 (26.3)	11 (31.4)	7 (19.4)	9 (22.5)	7 (15.9)	
Face and Neck	11 (4.3)	-	2 (4.8)	-	1 (2.9)	3 (8.3)	3 (7.5)	2 (4.5)	
Thorax	11 (4.3)	1 (4.3)	-	-	4 (11.4)	-	2 (5.0)	4 (9.1)	
Abdomen and pelvis	4 (1.6)	-	-	-	1 (2.9)	1 (2.8)	-	2 (4.5)	
Spine	6 (2.3)	1 (4.3)	-	-	3 (8.6)	-	2 (5.0)	-	
Upper extremities	114 (44.2)	2 (8.7)	11 (26.2)	18 (47.4)	13 (37.1)	22 (61.1)	22 (55.0)	26 (59.1)	
Lower extremities	47 (18.2)	4 (17.4)	7 (16.7)	7 (18.4)	8 (22.9)	4 (11.1)	7 (17.5)	10 (22.7)	
**Definitive Care Mode of Arrival, n (%)**									<0.001
Red Crescent ambulance	10 (3.9)	1 (4.3)	2 (4.8)	1 (2.6)	1 (2.9)	2 (5.7)	2 (5.0)	1 (2.3)	
Helicopter	3 (1.2)	2 (8.7)	-	-	-	-	1 (2.5)	-	
Govt/private ambulance	59 (22.9)	7 (30.4)	14 (33.3)	4 (10.5)	9 (25.7)	7 (19.4)	8 (20.0)	10 (22.7)	
Private/police vehicle	148 (57.4)	12 (52.2)	21 (50.0)	29 (76.3)	20 (57.1)	18 (50.0)	23 (57.5)	25 (56.8)	
Walking	13 (5.0)	-	2 (4.8)	2 (5.3)	3 (8.6)	2 (5.6)	2 (5.0)	2 (4.5)	
Unknown or not documented	25 (9.7)	1 (4.3)	3 (7.1)	2 (5.3)	2 (5.7)	7 (19.4)	4 (10.0)	6 (13.6)	
**Prior facility, *n* (%) $**									0.800
Yes	68 (26.8)	8 (36.4)	14 (34.1)	7 (18.4)	10 (29.4)	8 (22.2)	10 (25.0)	11 (25.6)	
No	186 (73.2)	14 (63.6)	27 (65.9)	31 (81.6)	24 (70.6)	28 (77.8)	30 (75.0)	32 (74.4)	
**Procedure at the scene, *n* (%)**									0.492
Yes	4 (1.6)	-	2 (4.8)	-	-	-	1 (2.5)	1 (2.3)	
No	254 (98.4)	23 (100.0)	40 (95.2)	38 (100.0)	38 (100.0)	36 (100.0)	39 (97.5)	43 (97.7)	
**Trauma Team Activation, *n* (%)**									0.235
Yes	7 (2.7)	-	-	1 (2.6)	3 (8.6)	1 (2.8)	-	2 (4.5)	
No	251 (97.3)	23 (100.0)	42 (100.0)	37 (97.4)	32 (91.4)	35 (97.2)	40 (100.0)	42 (95.5)	
**Blood transfusion in ED, *n* (%)**									0.283
Yes	7 (2.7)	-	1 (2.4)	-	2 (5.7)	-	3 (7.5)	1 (2.3)	
No	251 (97.3)	23 (100)	41 (97.6)	38 (100.0)	33 (94.3)	36 (100.0)	37 (92.5)	43 (97.7)	
**On arrival at the ED, mean (SD)**									
First systolic BP	89 (42.4)	107 (44.2)	86 (42.7)	72.8(49.2)	82 (45.1)	94 (36.0)	97 (34.6)	88 (42.9)	0.048
First heart rate	112 (21.3)	111 (27.7)	112 (27.7)	121 (20.7)	114 (18.2)	108 (14.2)	106 (16.5)	102 (15.5)	<0.001
First RR	22 (4.0)	23 (5.3)	23 (4.6)	23 (4.6)	22 (4.4)	21 (2.9)	20 (2.8)	20 (3.0)	0.003
First O_2_ saturation	97 (1.9)	96 (2.9)	96 (2.8)	98 (1.2)	98 (1.1)	98 (1.2)	97 (1.9)	98 (1.0)	<0.001

Note: Emergency Department = ED; Standard Deviations = SD; Blood Pressure = BP; Respiratory Rate = RR. ** Injury categories are not mutually exclusive, as some patients sustained injuries involving more than one anatomical region. Percentages were calculated using the total number of patients (n = 258) as the denominator; therefore, percentages exceed 100% when summed across injury categories. $ Prior facility indicates whether the patient was transferred from another healthcare facility before presentation to King Saud Medical City (Yes/No).

**Table 2:** Level of consciousness, injury severity, clinical management, and outcomes in paediatric fall-related injuries (N = 258).

Variables	All Patients	Age < 1	1 Year	2 Years	3 Years	4 Years	5 Years	6 Years	*p*-Value
	N = 258	N = 23	N = 42	N = 38	N = 35	N = 36	N = 40	N = 44	
**GCS score, *n* (%)**									<0.001
3−8	10 (3.9)	2 (8.7)	-	1 (2.6)	3 (8.6)	2 (5.6)	2 (5.0)	-	
9−12	6 (2.3)	1 (4.3)	2 (4.8)	-	1 (2.9)	1 (2.8)	1 (2.5)	-	
13−15	201 (77.9)	15 (65.2)	31 (73.8)	32 (84.2)	27 (77.1)	26 (72)	31 (77.5)	39 (88.6)	
Unknown or not documented	41 (15.9)	5 (21.7)	9 (21.4)	5 (13.2)	4 (11.4)	7 (19.4)	6 (15.0)	5 (11.4)	
**ISS, *n* (%)**									0.426
≤15	229 (88.8)	22 (95.7)	35 (83.3)	36 (94.7)	29 (82.9)	34 (94.4)	33 (82.5)	40 (90.9)	
16–25	23 (8.9)	-	5 (11.9)	2 (5.3)	4 (11.6)	2 (5.6)	6 (15.0)	4 (9.1)	
26–40	6 (2.3)	1 (4.3)	2 (4.8)	-	2 (5.7)	-	1 (2.5)	-	
**Disposition from ED, *n* (%)**									0.845
Ward	217 (84.1)	18 (78.3)	36 (85.7)	31 (81.6)	29 (82.9)	32 (88.8)	34 (85.0)	37 (84.1)	
ICU	29 (11.2)	5 (21.7)	3 (7.1)	5 (13.2)	5 (14.3)	3 (8.3)	3 (7.5)	5 (11.4)	
Operating theatre	12 (4.7)	-	3 (7.1)	2 (5.3)	1 (2.9)	1 (2.8)	3 (7.5)	2 (4.5)	
**Requires Operation, n (%)**									0.016
Yes	172 (66.7)	9 (39.1)	23 (54.8)	28 (73.7)	22 (62.9)	27 (75.0)	29 (72.5)	34 (77.3)	
No	86 (33.3)	14 (60.9)	19 (45.2)	10 (26.3)	13 (37.1)	9 (25.0)	11 (27.5)	10 (22.7)	
**Mechanical Ventilation, *n* (%)**									0.160
For ≤24 h	1 (0.4)	-	-	1 (2.6)	-	-	-	-	
For >24 and <96 h	8 (3.1)	2 (8.7)	2 (4.8)	2 (5.3)	-	-	2 (5.0)	-	
For ≥96 h	16 (6.2)	1 (4.3)	4 (9.5)	-	5 (14.3)	3 (8.3)	3 (7.5)	-	
No MV	233 (90.3)	20 (87.0)	36 (85.7)	35 (92.1)	30 (85.7)	33 (91.7)	35 (87.5)	44 (100)	
**ICU admission, *n* (%)**									0.900
Yes	39 (15.1)	5 (21.7)	7 (16.7)	5 (13.2)	6 (17.1)	4 (11.1)	7 (17.5)	5 (11.4)	
No	219 (84.9)	18 (78.3)	35 (83.3)	33 (86.8)	29 (82.9)	32 (88.9)	33 (82.5)	39 (88.6)	
**Days in ICU (in days)**									
Mean	1.3	1.2	1.1	1.1	1.1	1.1	1.1	1.1	
**Days in hospital (in days)**									
Median (IQR)	3.0 (3)	4.0 (12)	4.5 (6)	2.0 (2)	2.0 (2)	4.4 (2.0)	3 (4)	3 (2)	
Mean (SD)	5.7	11 (14.1)	7.3 (8.1)	3.5 (3.4)	5. 0 (5.8)	3 (5.0)	5.2 (6.4)	5.4 (7.8)	
**Hospital Discharge Destination *n* (%)**									0.723
Discharged home	253 (98.1)	23 (100)	42 (100)	37 (97.4)	35 (100.0)	34 (94.4)	38 (95.0)	44 (100)	
Transfer	2 (0.8)	-	-	-	-	1 (2.8)	1 (2.5)	-	
Mortuary/died	3 (1.2)	-	-	1 (2.6)	-	1 (2.8)	1 (2.5)	-	

Note: Glasgow Coma Scale = GCS; Injury Severity Score = ISS; Emergency Department = ED; Intensive Care Unit = ICU; Not MV = Not mechanically ventilated; standard deviations = SD; Interquartile Range = IQR.

**Table 3:** Univariate logistic regression model predicting Mechanical Ventilation and ICU admission.

Independent Variable	Mechanical Ventilation	ICU Admission
	OR (95% CI) *p*-Value	OR (95% CI) *p*-Value
**Age**		
3≤	0.48 (0.17–1.30) 0.166	0.9 (0.44–1.80) 0.796
4–6	0.58 (0.20–1.20) 0.133	0.7 (0.31–1.51) 0.457
**Gender**		
Male	Ref	Ref
Female	1.9 (0.80–4.50) 0.107	2.1 (1.08–4.21) 0.029
**Nationality**		
Non-Saudi	Ref	Ref
Saudi	1.1 (0.30–2.50) 0.802	1.5 (0.53–4.25) 0.378
**Injury time**		
Day	Ref	Ref
Night	1.3 (0.40–3.69) 0.589	1.5 (0.60–3.73) 0.310
**Day of week**		
Weekday (Sun–Thur)	Ref	Ref
Weekend (Fri–Sat)	1.0 (0.40–2.50) 0.991	0.8 (0.40–1.80) 0.732
**Injury cause**		
Fall–low (same level or <1 metre)	Ref	Ref
Fall–high (>1 metre)	2.8 (1.20–6.70) 0.016	2.7 (1.30–5.40) 0.005
**Injury Location**		
Home/house	1.4 (0.50–3.40) 0.443	1.3 (0.60–2.70) 0.421
**Injury type, *n* (%)**		
Head	23.7 (6.80–82.30) < 0.001	14.5 (6.20–33.80) < 0.001
Face and Neck	2.16 (0.40–10.60) 0.342	3.4 (0.90–12.46) 0.057
Thorax	9.4 (2.60–33.70) < 0.001	7.7 (2.20–26.90) < 0.001
Abdomen and pelvis	3.1 (0.30–31.90) 0.323	5.8 (0.80–42.90) 0.082
Spine	4.9 (0.80–28.60) 0.072	6.0 (1.10–30.80) 0.032
Upper extremities	0.29 (0.20–0.40) 0.002	0.08 (0.0–0.20) < 0.001
Lower extremities	0.40 (0.20–1.20) 0.086	0.2 (0.0–0.90) 0.036
**Definitive Care Mode of Arrival**		
Red Crescent ambulance	1.03 (1.20–8.50) 0.973	1.4 (0.20–6.91) 0.662
Helicopter	20.1 (1.70–231.0) 0.016	11.7 (1.0–133.22) 0.046
Govt/Private ambulance	7.8 (3.20–18.90) < 0.001	6.3 (3.0–13.13) < 0.001
Private/police vehicle	0.1 (0.05–0.40) < 0.001	0.2 (0.10–0.43) < 0.001
**Procedure at the scene**	0.3 (0.3–3.10) 0.323	0.1 (0.0–1.24) 0.082
**Blood transfusion in ED**	0.2 (0.0–1.30) 0.111	0.1 (0.0–0.55) 0.007
**Requires Operation**	5.0 (2.0–12.20) < 0.001	4.0 (1.90–8.23) < 0.001

Note: OR: odds ratio; ICU: intensive care unit.

**Table 4:** Multivariate regression model predicting Mechanical Ventilation and ICU admission.

Independent Variable	Mechanical Ventilation	ICU Admission
	AOR (95% CI) *p*-Value	AOR (95% CI) *p* Value
**Female**	*	3.13 (1.22–8.04) 0.018
**High Fall**	1.48 (0.51–4.27) 0.460	1.99 (0.78–5.05) 0.148
**Head Injury**	17.66 (3.42–91.03) < 0.001	6.41 (1.99–20.64) 0.002
**Thorax Injury**	13.36 (1.67–106.7) 0.014	5.19 (0.89–30.22) 0.067
**Spine Injury**	*	6.46 (0.72–57.75) 0.095
**Upper Extremities Injury**	0.58 (0.10–3.34) 0.550	0.21 (0.05–0.93) 0.040
**Lower Extremities Injury**	*	0.39 (0.06–2.47) 0.323
**MOA Helicopter**	11.98 (0.65–218.1) 0.094	5.73 (0.31–103.4) 0.236
**MA Private/Govt Ambulance**	3.71 (.70–19.71) 0.123	2.35 (0.57–9.57) 0.233
**MOA Private Police/Vehicle**	0.80 (0.13–4.85) 0.816	0.64 (0.15–2.65) 0.540
**Blood Transfusion in Emergency**	*	0.28 (0.03–2.41) 0.247

Note: * Was not significant in the univariate analysis; AOR = Adjusted odds ratios; MOA = Mode of Arrival.

## Data Availability

The datasets used during the current study are available from the corresponding author on reasonable request.
